# Distinct Genomic Profiles of Two Small Malignant Lesions Associated With an Intraductal Papillary Mucinous Neoplasm Co-occurring in a Patient

**DOI:** 10.7759/cureus.51394

**Published:** 2023-12-31

**Authors:** Hiroshi Ohyama, Yosuke Hirotsu, Hitoshi Mochizuki, Naoya Kato, Masao Omata

**Affiliations:** 1 Gastroenterology, Chiba University, Chiba, JPN; 2 Genome Analysis Center, Yamanashi Central Hospital, Kofu, JPN; 3 Gastroenterology, Yamanashi Central Hospital, Kofu, JPN

**Keywords:** pancreatic cancer, oncogenes, neoplasms, mutation, intra-ductal papillary neoplasm, genomics

## Abstract

Intraductal papillary mucinous neoplasm of the pancreas (IPMN) is characterized by cystic dilatation of the pancreatic duct system, intraductal papillary growth, and excessive mucin secretion. Although IPMN is basically a benign disease and surgical resection is not necessary, it has the potential to develop into pancreatic cancer. We recently encountered a rare case of synchronous development of two different types of malignant lesions in the pancreas associated with IPMN derived from different clones. A 74-year-old Japanese woman developed a cystic lesion in her pancreatic tail. Endoscopic ultrasound-guided fine needle aspiration (EUS-FNA) was performed on two low echoic lesions in the pancreatic tail (10 mm) and body (10 mm), which were then diagnosed as malignancies. After the surgically resected pancreas was carefully examined, in addition to the tail (10 mm) and body (10 mm) tumors, an intraductal papillary mucinous adenoma (IPMA) was observed, which was continuous to the tail tumor and extending toward the body of the pancreas but not contiguous to the body tumor. Genomic analysis using targeted sequencing revealed that the malignant lesion in the pancreatic tail and two sections of adjacent IPMA lesions in the pancreatic duct were almost identical. KRAS G12D, RNF43 G29fs, PBRM1 P1471R, and PIK3CA I1058L were shared, whereas only KRAS G12D was shared between the malignant lesion in the pancreatic body and others. Multiple pancreatic cancers may occur simultaneously and/or metachronously in the context of genomic alterations in IPMN.

## Introduction

Intraductal papillary mucinous neoplasm of the pancreas (IPMN) is characterized by cystic dilatation of the pancreatic duct system, intraductal papillary growth, and excessive mucin secretion [1]. Intraductal papillary mucinous neoplasm of the pancreas has been categorized into main-duct IPMN (MD-IPMN) and branch-duct IPMN (BD-IPMN) based on the location of the involved pancreatic duct and the presence of cystic dilatation of the branch ducts [2]. Surgical resection is generally warranted for MD-IPMN due to a high malignancy rate of >60% [3]. Conversely, because approximately 80% of resected BD-IPMNs are benign without histopathological features of high-grade intraepithelial neoplasia or invasive tumor growth, BD-IPMNs are primarily thought to be benign tumors [3]. However, there is a relevant risk of malignant transformation over time [4]. We recently encountered a rare case of two synchronous malignant lesions in the pancreatic body and tail associated with BD-IPMN. We present a detailed analysis of the genomic profiles of the two tumors and their associated adenoma.

## Case presentation

A 74-year-old woman without any symptoms was determined to have abnormal liver enzyme levels by a primary care physician. She underwent abdominal computed tomography (CT) and magnetic resonance cholangiopancreatography (MRCP), and a cystic lesion of the pancreas measuring 30 mm in the tail was detected. Because this cystic lesion was regarded as BD-IPMN with alarming features (cyst ≥30 mm), she was referred to our hospital for examination of the lesion by endoscopic ultrasonography (EUS) and EUS-guided fine needle aspiration (EUS-FNA).

**Figure 1:**
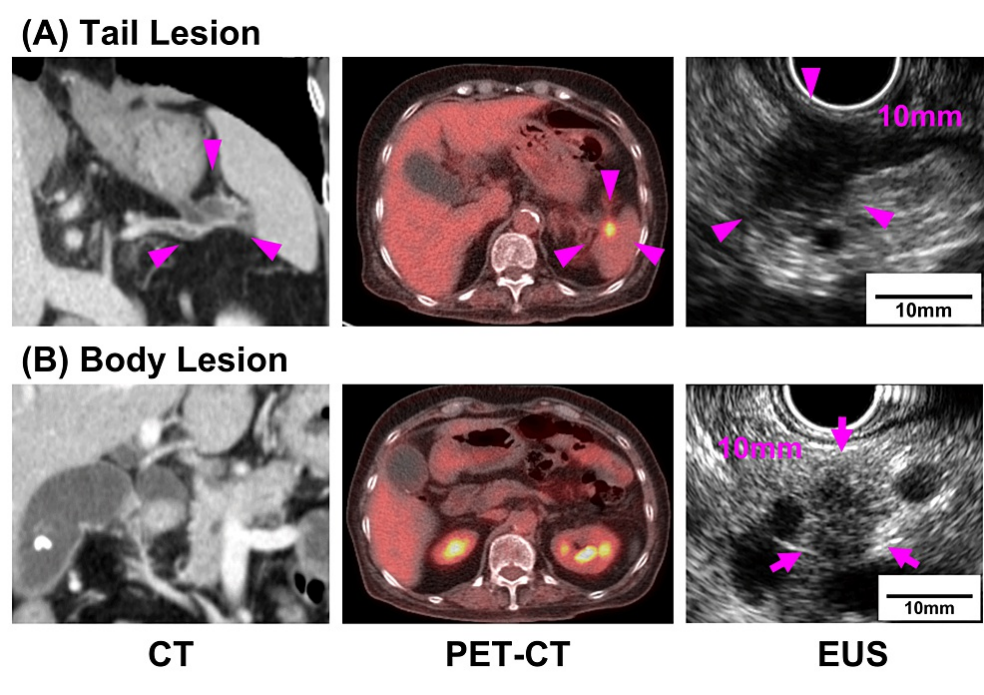
Two lesions in the pancreatic tail and body on CT, PET-CT, and rechecked EUS imaging. The lesion of the pancreatic tail (arrowhead) was detected by all imaging (A). However, the lesion of the pancreatic body (arrow) was detected only by EUS (B). CT: computed tomography; EUS: endoscopic ultrasound; PET: positron emission tomography

As an outpatient, EUS was performed for the patient, and a cystic lesion (16 mm) in the pancreatic tail was detected and diagnosed as BD-IPMN. Moreover, a faint low echoic lesion (10 mm) without main pancreatic duct dilatation was found in the pancreatic body (Figure 1).

**Figure 2:**
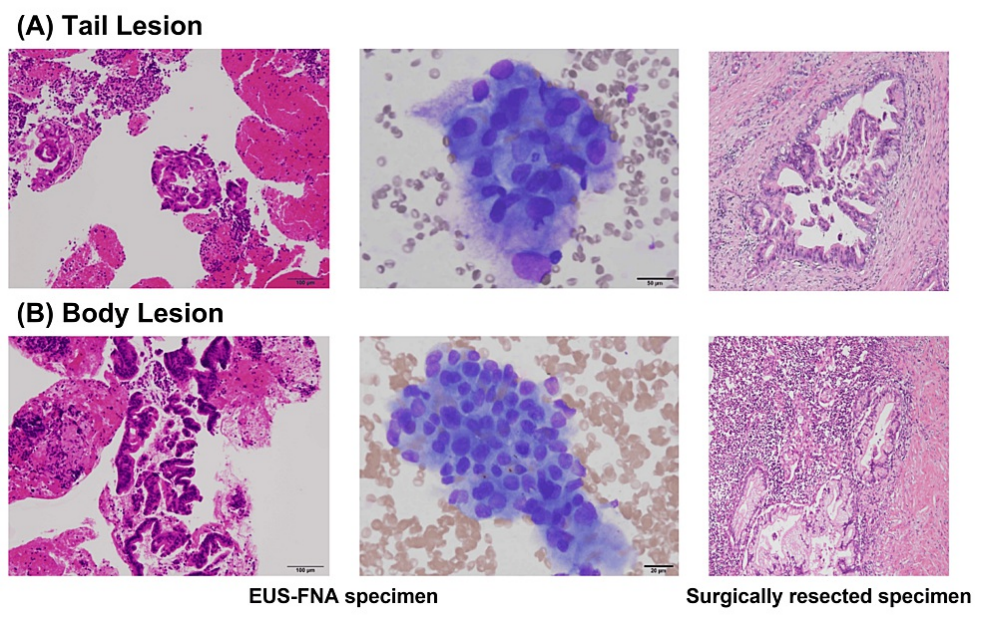
Photomicrographs of histological and cytological imaging obtained by EUS-FNA and surgery. The imaging of histology and cytology from the lesions of the pancreatic tail (A) and body (B) was obtained by EUS-FNA and surgery. The pathological findings in the pancreatic body and tail were invasive ductal carcinoma, well-to-moderately differentiated adenocarcinoma (tub1>tub2), and intraductal papillary mucinous neoplasm with focal invasive adenocarcinoma, respectively. EUS-FNA: endoscopic ultrasound-guided fine needle aspiration

Positron-emission tomography with CT (PET-CT) was subsequently performed, and abnormal accumulation of 18F-fluorodeoxyglucose was observed (SUVmax 8.37) in the lesion of the pancreatic tail (Figure 1A) but not in the lesion of the pancreatic body (Figure 1B). Therefore, the EUS of the lesion of the pancreatic tail was repeated, and the EUS-FNA of the two lesions was subsequently performed. The histological and cytological findings of the EUS-FNA specimens from the pancreatic tail were adenocarcinoma and class IV, respectively, and those from the pancreatic body were suspicious of IPMN and class IV, respectively (Figure 2).

**Figure 3:**
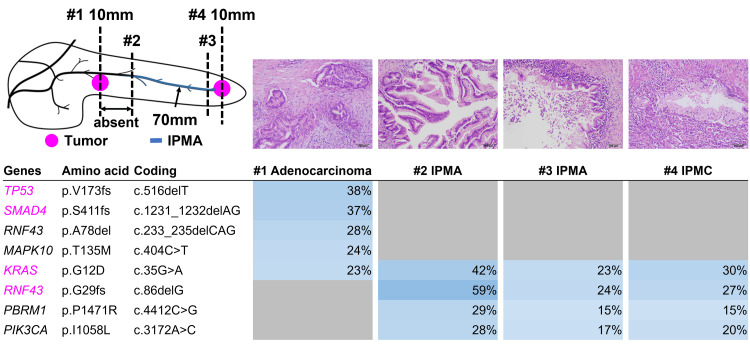
Genomic profiles of the two malignant and two IPMA lesions. Upper panel: Location of lesions (pink dot: tumor, blue line: IPMA). Lesions #1 and #4 were malignant, and lesions #2 and #3 were diagnosed as IPMA. Photomicrographs corresponding to the lesions were indicated. Original magnification: 100×, scale bar: 100 μm. Lower panel: heat map of the mutation profile in each sample. The left column lists the names of mutated genes with the corresponding amino acid changes. Pink letters indicate oncogenic mutations annotated by the OncoKB database. Variant allele fraction is shown in each box and is indicated by the graduated color scale from 1% (light blue) to 100% (dark blue). The gray box indicates no identified mutations. EUS-FNA: endoscopic ultrasound-guided fine needle aspiration; FFPE: formalin-fixed paraffin-embedded; IPMA: intraductal papillary mucinous adenoma; IPMC: intraductal papillary mucinous carcinoma.

The patient underwent a distal pancreatectomy at our hospital. The postoperative hospital stay was uneventful. The lesion in the tail from surgically resected specimens was histologically diagnosed as IPMN with focal invasive adenocarcinoma (pathological stage IIA), and that in the body as well to moderately differentiated adenocarcinoma (pathological stage IA) (Figure 2). The IPMN lesion was contiguous with the tumor in the pancreatic tail but was 10 mm discontinuous with the tumor in the pancreatic body. Moreover, although preoperative multiple imaging, including CT, MRCP, PET-CT, and EUS, did not detect the change in the pancreatic duct, the IPMN lesion was extended along the pancreatic duct (Figure 3).

Next, a genomic profile analysis was conducted. We performed targeted sequencing for formalin-fixed, paraffin-embedded (FFPE) tissues using next-generation sequencing with an internally generated panel of 60 significantly mutated genes (280,220 base pairs), and we were repeatedly shown to cover all significantly mutated genes, including key driver genes in pancreaticobiliary cancer (Table 1) [5, 6].

**Table 1:** Genes assayed in the pancreaticobiliary cancer panel DNA: deoxyribonucleic acid; MAPK: mitogen-activated protein kinase; NIDPH: nicotinamide adenine dinucleotide phosphate; PI3K: phosphoinositide 3-kinases; SMG: significantly mutated gene; TGFβ: transforming growth factor beta

Gene List (Pancreaticobiliary Cancer Panel, 60 SMGs)									
Signaling Pathways	Genes								
MAPK	BRAF	EGFR	ERBB2	ERBB3	ERBB4	FGFR2	GNAS	HRAS	JAK3
	KRAS	MAP2K4	MAP2K7	MAPK10	NF1	NRAS	NRG1	SOS2	SRC
Epigenetic	ARID1A	ARID1B	ARID2	BAP1	EPC1	KMT2A	KMT2C	PBRM1	
PI3K	AKT1	PIK3CA	PTEN	STK11	TSC1	TSC2			
DNA Damage Repair	ATM	BRCA1	BRCA2	CDKN2A	PALB2	TP53			
TGFβ	ACVR1B	ACVR2A	SMAD4	TGFBR2					
Wnt	APC	AXIN1	CTNNB1	RNF43					
Mismatch Repair	MLH1	MSH2	MSH6	PMS2					
Axon Guidance	ROBO1	ROBO2	SLIT2						
Transcriptional Activator	ELF3	NFE2L2							
Splicing Factors	RBM10	SF3B1							
NADPH Metabolism	IDH1	IDH2							
Myc	MYC								

Since the lesions were rather small and tumor cells are often embedded in dense fibrotic tissues, laser capture microdissection was carefully performed to obtain neoplastic tissues using an Arcturus XT Laser Capture Microdissection System (Thermo Fisher Scientific, Waltham, MA). After the surgically resected pancreas was carefully examined, in addition to two tail (10 mm) and body (10 mm) tumors, an intraductal lesion was observed, which was continuous to the tail tumor and extending toward the body of the pancreas but not contiguous to the body tumor. We analyzed four FFPE sections (body tumor, #2 and #3 sections from the intraductal lesion, and tail tumor) (Figure 2). The malignant lesion in the pancreatic tail and two sections of adjacent intraductal lesions in the pancreatic duct were almost identical. KRAS G12D, RNF43 G29fs, PBRM1 P1471R, and PIK3CA I1058L were shared, whereas only KRAS G12D was shared between the malignant lesion in the pancreatic body and others (Figure 2). After surgical resection, her liver function improved, and the patient was regularly evaluated for cancer recurrence with blood tests and CT imaging. Eight months after surgical resection, multiple pulmonary nodules appeared and were diagnosed as lung metastases.

## Discussion

Intraductal papillary mucinous neoplasm of the pancreas has been recognized as a precursor lesion to pancreatic carcinoma [1]. In a large long-term study on patients with BD-IPMN, the five-year incidence rate of pancreatic malignancy was 3.3%, reaching 15.0% 15 years after IPMN diagnosis [4]. Although BD-IPMN has a low risk of malignancy, in this case, two early-stage malignant lesions separated in the body and tail were detected synchronously and successfully treated. Furthermore, careful examination of the resected specimen revealed the "third” lesion, which is confirmed from the tail mass to the body lesion. Only the continuity of the tail lesion was confirmed. The association between these lesions could be speculated in many ways.

Surprisingly, intraductal lesions could not be identified preoperatively and became apparent postoperatively. Although it is unclear whether the intraductal lesion or tumor in the pancreatic tail developed first, the continuity of the lesions and identical genomic profile suggests that they are identical in origin. Conversely, the body lesions are distant from the other two lesions and share only KRAS in their genomic profile, which is likely to be of a different origin since KRAS is a mutation that is highly prevalent in pancreatic cancer. Recently, comprehensive genomic study data revealed the molecular profiles of pancreatic cancer and provided a catalog of somatic mutations [5, 7]. Previous studies revealed that KRAS, GNAS, and RNF43 were frequently mutated in IPMN [8-10]. The results of this study indicated that both malignant lesions were derived from IPMN because of the presence of KRAS and RNF43 mutations, but different types of oncogenic evolution were presumed to have occurred in each lesion.

## Conclusions

In conclusion, we encountered an extremely rare case of two simultaneous pancreatic cancers in the background of IPMN. Genomic analysis of the two malignant lesions, including the adjacent intraductal papillary mucinous adenoma, revealed that one developed pancreatic cancer derived from IPMN, and the other developed pancreatic cancer from a different evolutionary lineage. Although IPMNs are a high-risk group of pancreatic cancer and require careful follow-up, it should be noted that multiple pancreatic cancers may occur simultaneously and/or metachronously in the context of genomic alterations of IPMN.
